# Understanding the role of e-cigarette use in smoking cessation based on the stages of change model

**DOI:** 10.1371/journal.pone.0274311

**Published:** 2022-09-09

**Authors:** Wonjeong Yoon, Inhyung Cho, Sung-il Cho

**Affiliations:** 1 Department of Public Health Science, Graduate School of Public Health, Seoul National University, Seoul, Republic of Korea; 2 Institute of Health and Environment, Seoul National University, Seoul, Republic of Korea; Stanford University, UNITED STATES

## Abstract

**Objective:**

We explored the role of e-cigarette use in smoking cessation based on the stages of change (SOC) model, which is a framework for describing the process of smoking cessation.

**Methods:**

We used nationwide, cross-sectional data on adults (19+ years) from the seventh Korea National Health and Nutrition Examination Survey (KNHANES, 2016–2018) and restricted the participants to 3,929 recent smokers, consisting of current smokers and recent quitters (≤2 years). A multinomial logistic regression analysis was performed to reveal the relationships between e-cigarette use and cigarette quitting behaviors (e.g., current quitting status, past quit attempts, intention to quit, and duration of quitting) and all stages in smoking cessation, with adjustment for sociodemographic and smoking-related factors.

**Results:**

E-cigarette use was positively related to past quit attempts, while not having quit, intention to quit, and longer duration of quitting. Based on the cessation stages, current and former e-cigarette users were significantly more likely to be in the ‘Precontemplation’ and ‘Contemplation’ stages than never users, while not to be in the ‘Preparation’ and ‘Action’ stages. Current users were particularly less likely to be in the ‘Maintenance’ stage compared to never users.

**Conclusion:**

E-cigarette use was closely linked with early-stage behavior than late-stage behavior in the smoking cessation process. E-cigarettes might promote quit attempts and short-term quitting in some smokers, but the negative role of inducing smokers to continue cigarette smoking with no immediate quit-intention for future attempts is dominant in the real world.

## Introduction

Electronic cigarette (e-cigarette) use has risen rapidly over the past decade. The number of e-cigarette users worldwide increased more than fivefold, from 7 million in 2011 to 41 million in 2018 [1]. The global sales of e-cigarettes reached 20 billion in 2021 compared to 2 billion in 2012 [2, 3].

With the growing popularity of e-cigarettes, their efficacy in substituting for cigarette smoking has emerged as a major public health concern [4–6]. E-cigarettes were initially promoted as smoking cessation tools [7], and so the most common reasons for using e-cigarettes by adults are related to cigarette quitting behaviors, such as to quit smoking or to manage withdrawal symptoms [8, 9]. However, claims about the positive and negative effects of e-cigarettes use on quitting smoking are still in conflict [4]. For example, some experts suggest that e-cigarettes can help smokers quit smoking and maintain abstinence [10–12] and may be more effective than conventional nicotine products [10, 13]. By contrast, other experts warn that e-cigarettes do not contribute to smoking cessation [14–16], and may rather delay or impede smoking cessation―by curbing the use of proven cessation methods or sustaining a nicotine addiction during periods of abstinence [17]. Further efforts are needed to in-depth understand the role of e-cigarette use in smoking cessation.

Smoking cessation outcomes are often assessed in a binary manner (i.e., cessation or not), focusing on the transition from current to former smokers [18, 19]. However, in principle, smoking cessation is not a single event, but a dynamic process involving a series of behavioral changes [20]. According to the stages of change (SOC) model, which is a theoretical framework for smoking cessation behavior, a smoker’s status toward quitting smoking consists of five motivational stages: pre-contemplation (PC), contemplation (C), preparation (P), action (A), and maintenance (M) [21, 22]. Each stage is determined by quitting behavioral factors, including current behavior, past quit attempts, intention to quit, and duration of quitting, and changes in these factors can cause progression or regression [23]. Although its conceptual validity has been questioned by some scholars [24–26], this model has the unique advantage of being sensitive to all changes in the smoking cessation process, which is not possible using traditional dichotomous cessation outcomes [18, 19].

Despite its potential utility, few studies have considered the SOC model in relation to e-cigarette use. Moreover, to the best of our knowledge, no previous study has covered all five stages of the model; most have considered three [27–29] or four [30, 31] stages. In this study, we extend the scope of previous studies to the full stages of the SOC model and explored the relationship between e-cigarette use and smoking cessation. Specific objectives were: 1) to examine the associations between e-cigarette use and cigarette quitting behavioral factors (i.e., current behavior, past quit attempts, intention to quit, and the duration of quitting) involved in cessation stages, and 2) to examine the relationship between the e-cigarette use and each cessation stage. The results provide insight into the role of e-cigarette use in the entire process of smoking cessation and facilitate the use of the SOC model in research on the real-world effectiveness of e-cigarette use.

## Methods

### Data and subjects

This study used a pooled dataset from the seventh Korea National Health and Nutrition Examination Survey (KNHANES) from 2016 to 2018. The KNHANES is a nationwide cross-sectional survey conducted by the Korea Centers for Disease Control and Prevention (KCDC). This survey collects health-related information on Koreans using health behavior surveys, health examinations, and a nutritional survey [32]. Interviews were conducted by trained medical staff and health interviewers. A representative sample was selected using stratified multistage probability sampling from 192 survey districts in Korea [32]. Of the participants (*n* = 24,269) in the seventh KNHANES, 19,389 adults (≥19 years old) were eligible for this study. Participants who responded “don’t know” or “refused” (n = 1,139) for main study factors such as e-cigarette use status, smoking status, quitting behaviors and sociodemographic characteristics were excluded. Of the remaining 18,250 respondents, the analytic sample was restricted to 3,929 recent smokers to examine the relationship between e-cigarette use and the stages of change in smoking cessation. They included current smokers defined as those who had smoked ≥ 100 cigarettes in their lifetimes and reported currently smoking “every day” or “some days”, and former smokers defined as those who had smoked ≥ 100 cigarettes in their lifetimes but reported currently smoking “not at all” and had stopped smoking in the past two years (hereafter referred to as ‘recent quitters’).

We defined recent quitters by the cut-off value of 2 years since quitting as in some previous studies by Li and by Gravely, et al [33, 34]. Cut-off with longer period will include more individuals in the maintenance stage. However, e-cigarettes are relatively new products and the cut-off should not be too long. Empirical evidence shows that 60–90% of smoking relapse in quitters occurred within the first year since quitting, and 15% of the rest (about 80% of total relapses) in the second year [35, 36]. Therefore, we considered 2 years cut-off would capture most of the quitting behaviors. Our data also identified that about 82.0% of current e-cigarette users and 61.9% of former e-cigarette users were concentrated among quitters in those who stopped smoking within the last 2 years (S1 Fig).

### Variables

#### Basic characteristics

The sociodemographic variables under study were sex (male, female), age group (19–34, 35–49, ≥50 years), educational level (college or more, high school, less than high school), income level (high, top 25%; middle; low, bottom 25%), and employment status (yes, no). Cigarette smoking characteristics included amount smoked (≥21, 11–20, 0–10 cigarettes/day) and age of smoking initiation (<19, ≥19 years). These factors were treated as categorical variables in the analysis.

#### E-cigarette use

E-cigarette use was assessed using the following two questions: “Have you ever used an e-cigarette in your lifetime?” and “Have you used an e-cigarette in the last month?” Respondents were classified into: “never e-cigarette users” who answered “no” to the first question; “current e-cigarette users” who answered “yes” to the first question and “yes” to the second question; and “former e-cigarette users” who answered “no” to the second question.

#### Cigarette quitting behaviors

Four cessation-related behavioral factors were used to measure the SOC: current behavior, past-year quit attempts, intention to quit, and duration of quitting. Current behavior was dichotomized into current smoking (i.e., current smoker) and non-current smoking (i.e., recent quitter). Past quit attempts were assessed among current smokers using the question, “In the last year, have you ever stopped smoking for at least 24 hours, because you were trying to quit? (yes or no).” The intention to quit smoking was measured in current smokers using the question, “Are you planning to quit smoking? (within the next month, within the next 6 months, sometime in the future beyond 6 months, or no plan to quit).” The duration of quitting was investigated in recent quitters using the question, “How long has it been since you quit smoking (monthly unit responses)?”

#### Stages of change in smoking cessation

The stages of change in smoking cessation was the main outcome variable. Based on the above four quitting behavioral factors, the traditional SOC in smoking cessation was classified into the following five stages: 1) Precontemplation (PC): current smokers who are not planning on quitting within the next 6 months; 2) Contemplation (C): current smokers who are planning on quitting within the next 6 months; 3) Preparation (P): current smokers who are both planning on quitting within the next month and have quit attempts in the past year; 4) Action (A): recent quitters who quit within the last 6 months; 5) Maintenance (M): recent quitters who quit more than 6 months ago [21, 22]. However, this traditional SOC model has the known problem that individuals in the PC and C stages are heterogeneous according to whether they made a quit attempt in the past year [23, 37]. Complementing this issue, this study separated the individuals who had not made a quit attempt in the past year from the PC and C stages and classified them as the ‘No attempt’ (NA) stage. A detailed classification of this extended SOC model is presented as a flowchart in S2 Fig.

**Fig 1:**
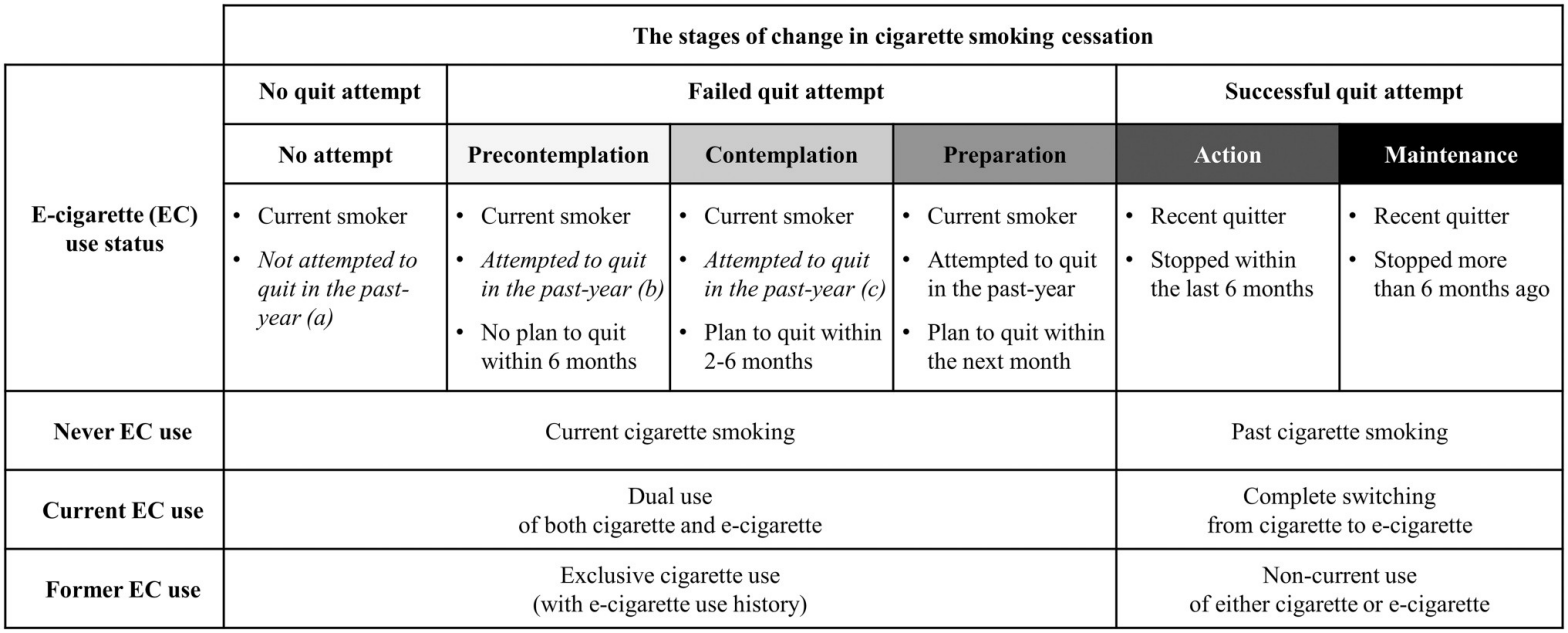
Modified* stages of change model in smoking cessation linked with e-cigarette use status. *Modification (in *italics*) from the original model was made by excluding those with no attempt (a) from the ‘Precontemplation’ and ‘Contemplation’ stages, leaving only those with failed quit attempt in these two stages (b and c). Stages are shaded darker for more progressed cessation stages.

In this SOC model extended to six stages along with the NA stage, individuals in the PC, C, and P stages are defined as current smokers who recently attempted to quit smoking. Notably, past quit attempts reported by current smokers in themselves imply that the respondent’s recent attempt to quit smoking was unsuccessful [38]. Specifying this characteristic in the process of quitting smoking makes it possible: 1) to distinguish ‘failed quitting attempts’ from ‘no attempt’ and ‘successful quit’, and 2) to compare the intentional status after failed attempts in parallel. Therefore, the expanded-classification of the SOC model reflecting past quit attempts will be useful in understanding and interpreting the role of e-cigarette use in smoking cessation. The specific pattern of e-cigarette use (e.g., dual use, complete switching, and non-current use) across the extended stages of smoking cessation are summarized in the Fig 1.

### Statistical analysis

We applied sampling weights to account for the complex sampling design of the survey [39]. Frequency analysis was performed with weighted percentages (%) to describe the distribution of samples according to e-cigarette use status. The chi-square test was performed to test the bivariate associations of e-cigarette use with cessation-related behavioral factors and the cessation stages, with a significance level of *P* < 0.05. A multivariate logistic regression analysis was conducted to explore the associations between e-cigarette use and cessation outcomes, adjusting for sociodemographic variables and smoking characteristics. The survey year was also included as a covariate because the rates of e-cigarette use may differ significantly according to the survey year. The results are presented as odds ratios (ORs) with 95% confidence intervals (CIs). Statistical analyses were conducted using SAS ver. 9.4. software (Proc Surveyfreq and Proc Surveylogistic).

### Ethics

The Seoul National University Institutional Review Board approved this study (IRB No. E2103/001-003).

## Results

### Sample characteristics by e-cigarette use status

Table 1 shows the sample characteristics according to e-cigarette use status. The participants included 353 (10.1%) current users, 881 (24.8%) former users, and 2695 (65.0%) never users. There were significant differences among these three groups in sociodemographic variables and smoking characteristics (*P* < 0.05). Regarding sociodemographic variables, current and former e-cigarette users tended to be male, younger, have higher education and income levels, and be employed. Concerning smoking characteristics, former e-cigarette users engaged in heavy smoking. E-cigarette users noted that they started smoking at a younger age.

**Table 1:** Demographics and smoking characteristics, overall and by e-cigarette use status.

	Overall		Current EC user		Former EC user		Never EC user		
	(N = 3,929)		(N = 353)		(N = 881)		(N = 2,695)		
	N	%	N	%	N	%	N	%	*P*
Sex									
Male	3290	85.9	308	88.6	752	87.5	2230	84.8	0.050^†^
Female	639	14.1	45	11.4	129	12.4	465	15.2	
Age group (years)									
19–34	902	30.6	137	45.1	358	48.5	407	21.5	<0.001
35–49	1388	36.6	153	41.1	345	37.0	890	35.8	
50+	1639	32.8	63	13.8	178	14.5	1398	42.8	
Education level									
College or more	1400	38.2	175	47.6	362	41.5	863	35.5	<0.001
High school	1477	40.5	139	41.9	377	46.0	961	38.2	
Less than high school	1052	21.3	39	10.4	142	12.5	871	26.3	
Income level									
High	988	26.6	122	35.4	238	26.3	628	25.3	<0.001
Moderate	1965	52.6	187	53.3	475	57.0	1303	50.8	
Low	976	20.8	44	11.4	168	16.7	764	23.9	
Employment				;.					
Yes	2785	73.3	282	81.2	666	76.3	1837	70.9	<0.001
No	1144	26.7	71	18.8	215	23.7	858	29.1	
Amount smoked^a^ (cigarettes/day)									
21+	272	6.8	24	6.4	72	7.4	176	6.7	0.018
11–20	1756	45.4	166	46.4	439	50.3	1151	43.3	
0–10	1901	47.8	163	47.3	370	42.3	1368	50.0	
Age started smoking (years)									
<19	1696	46.4	195	57.0	469	55.1	1032	41.4	<0.001
≥19	2229	53.6	158	43.0	412	44.9	1659	58.6	
E-cigarette use status									
Current use	353	10.1							
Former use	881	24.8							
Never use	2695	65.0							

Values are unweighted numbers (N), weighted percentages (%), and P-values by chi-square test.

The significance of P-value is set at a level of 0.05.

^†^ The P-value was marginally significant (*P* = 0.0499).

^a^ Amount smoked for quitters was assessed retrospectively.

EC = e-cigarette.

### Prevalence of quitting behaviors and cessation stages

Table 2 shows the prevalence of quitting behaviors and cessation stages among all participants. The participants included 3,323 (85.1%) current smokers and 606 (14.9%) recent quitters. Of current smokers, more than half (56.0%) have made quit attempts in the past year and about one-third (33.5%) planned to quit smoking within the next 6 months. Of recent quitters, nearly two-third (62.4%) reported quitting for longer than 6 months. Based on the stages of change in smoking cessation, majority (62.3%) of overall participants were in NA or PC stage, and followed by P (13.2%), C (9.7%), M (9.3%) and A (5.6%), stages.

**Table 2:** Prevalence of quitting behaviors and cessation stages among all participants (N = 3,929).

	N	%
Current behavior		
Current smoker	3323	85.1
Recent quitter (≤2 years)	606	14.9
Past-year quit attempt^a^		
No	1461	44.0
Yes	1862	56.0
Intention to quit^a^		
Not within 6 months	2235	66.5
Within 2–6 months	456	14.8
Within 1 month	632	18.7
Duration of quitting^b^		
≤6 months	229	37.6
7–24 months	377	62.4
Stages of change		
No attempt	1461	37.5
Precontemplation	984	24.8
Contemplation	350	9.7
Preparation	528	13.2
Action	229	5.6
Maintenance	377	9.3

Values are unweighted numbers (N) and weighted percentages (%).

^a^ Only asked of current smokers (n = 3,323),

^b^ Only asked of recent quitters (n = 606).

### Associations between e-cigarette use and quitting behavioral factors

#### Current behavior

Table 3 presents the prevalence and odds ratio of cessation outcomes by e-cigarette use status. Current quitting behavior did not significantly differ by e-cigarette use stats (*P* = 0.2106). 11.5% of current users being quitters and 14.6% and 15.5% of former and never users, respectively. Multivariate regression analysis showed that current e-cigarette users were significantly less likely to be quitters (aOR = 0.64, 95% CI = 0.41–0.98) than never users. Former e-cigarette users also showed lower likelihood of being quitters than never users (aOR = 0.86, 95% CI = 0.66–1.12), but not statistically significant.

**Table 3:** Multivariate associations between e-cigarette use and cigarette quitting characteristics.

	Being quitter			Past quit attempt^a^			Intention to quit^a^			Long-term quitting^b^		
	%	aOR	(95% CI)	%	aOR	(95% CI)	%	aOR	(95% CI)	%	aOR	(95% CI)
E-cigarette use status												
Current EC user	11.5	0.64*	(0.41–0.98)	60.7	**1.44** *	**(1.08–1.92)**	18.3	1.06	(0.72–1.57)	32.1	**0.20** ***	**(0.09–0.43)**
Former EC user	14.6	0.86	(0.66–1.12)	59.3	**1.35** **	**(1.11–1.65)**	15.6	0.86	(0.66–1.12)	63.9	0.87	(0.53–1.45)
Never EC user	15.5	1.00	Ref.	53.9	1.00	Ref.	19.9	1.00	Ref.	65.4	1.00	Ref.
	*P* = 0.2106			*P* = 0.0199			*P* = 0.0739			*P* = 0.0004		

Values are weighted percentages (%), adjusted odds ratios, 95% confidential intervals, and P-values according to chi-square test.

Multivariable models were adjusted for all covariates in Table 1 and the survey year.

^a^ Only asked of current smokers (n = 3,323),

^b^ Only asked of recent quitters (n = 606).

* P < 0.05;

** P < 0.01;

*** P < 0.001;

Bold type = P < 0.05.

aOR = adjusted odds ratio; CI = confidence interval; EC = e-cigarette.

#### Past-year quit attempts

The percentages of past quit attempts differed by e-cigarette use status (*P* = 0.0199) (Table 3). About 61% of current e-cigarette users and 59% of former users had quit attempts in the past year, compared to 54% of never users. This difference remained in a multivariate regression analysis adjusting for covariates. Current and former e-cigarette users were more likely to report prior attempts to quit smoking (current: aOR = 1.44, 95% CI = 1.08–1.92; former: aOR = 1.35, 95% CI = 1.11–1.65) than never users.

#### Intention to quit smoking

There were no significant differences in intention to quit within 1 month by e-cigarette use status (*P* = 0.0739) (Table 3). About one-fifth (19.9%) of never users planned to quit within the next month, and 18.3% and 15.6% of current and former users, for each. In a multivariate analysis controlling for covariates, current e-cigarette users were non-significantly more likely to report intention to quit smoking within the next month (current: aOR = 1.06, 95% CI = 0.72–1.57) than never users. Former e-cigarette users even reported negative results (aOR = 0.86, 95% CI = 0.66–1.12) for intention to quit compared to never users, although it is not significant.

#### Duration of quitting

According to e-cigarette use status, current users showed a significantly lower proportion (32.1%) of long-term abstinence compared to former users (63.9%) and never users (65.4%) (*P* = 0.0004) (Table 3). This difference remained after adjusting for covariates. Current users were less likely to maintain smoking abstinence for >6 months (aOR = 0.20, 95% CI = 0.09–0.43) than never users. Former users did not significantly differ from never users in terms of long-term abstinence (aOR = 0.87, 95% CI = 0.53–1.45).

### Association between e-cigarette use and cessation stages

**Fig 2:**
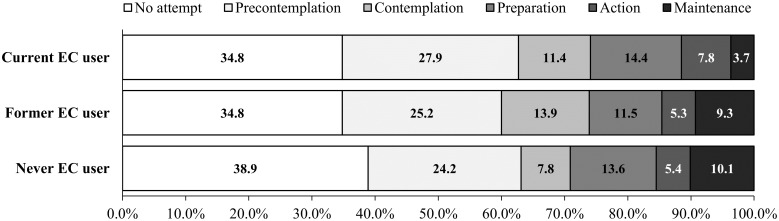
Prevalence of the stages of change in cigarette smoking cessation by e-cigarette use status. This is a stacked bar graph showing the distribution of percentages of cessation stages among current (n = 353), former (n = 881), and never (n = 2,695) e-cigarette users. EC = e-cigarette.

Fig 2 shows the prevalence of stages of change in cigarette smoking cessation among current, former, and never e-cigarette users. Overall, the distribution of the cessation stages varied by the e-cigarette use status (P < 0.001) (S1 Table). Current e-cigarette users had higher proportions of the PC (27.9%), C (11.4%), P (14.4%) and A (7.8%) stages than never users (24.2%, 7.8%, 13.6%, and 5.4%, for each), and former e-cigarette users had higher proportions of the PC (25.2%), and C (13.9%) stages than never users (24.2%, and 7.8%, respectively). Never e-cigarette users had higher proportions of the M (10.1%) stages than current and former e-cigarette users (3.7% and 9.3%, for each).

**Fig 3:**
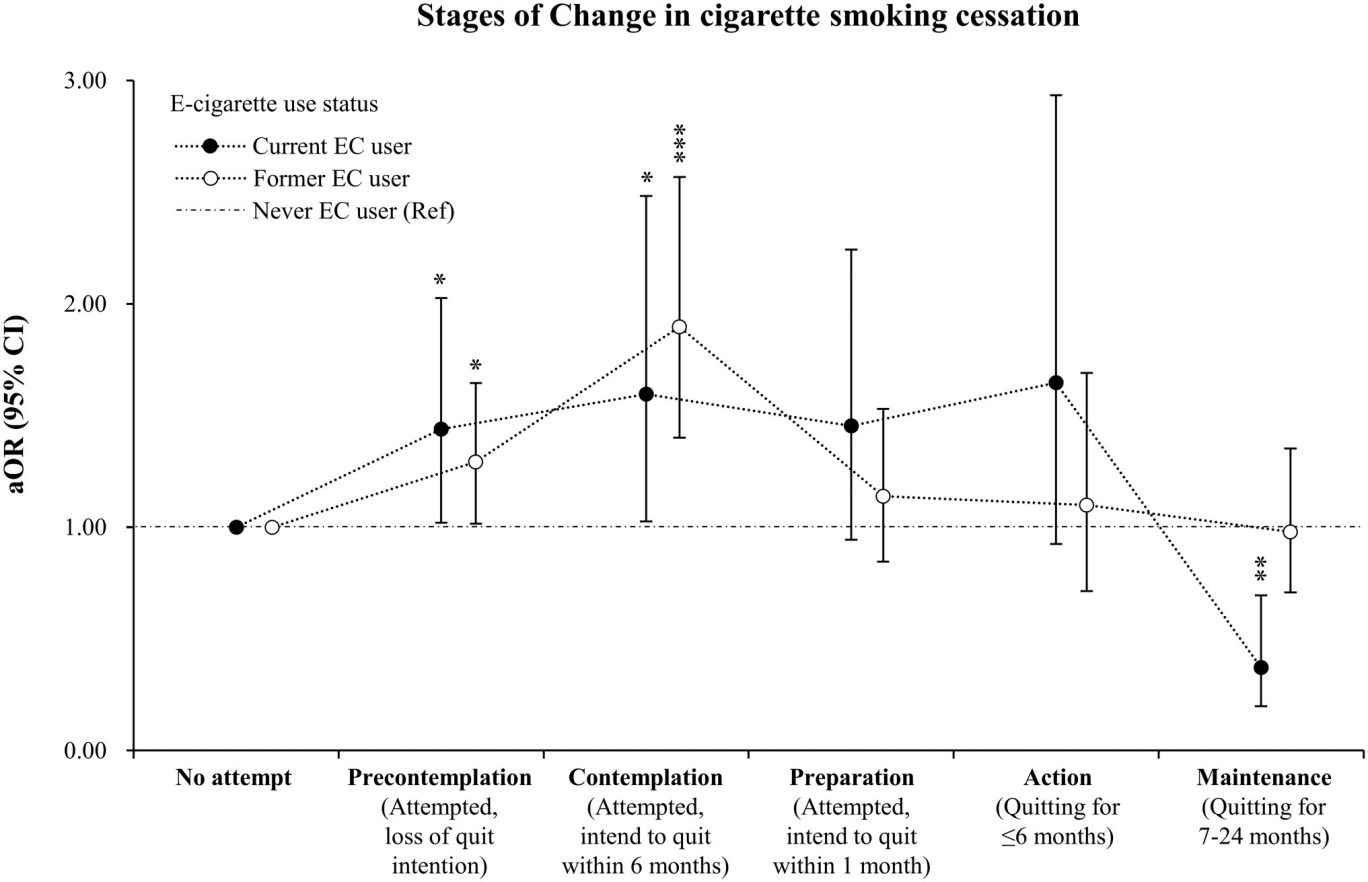
Odds ratio for the stages of change in cigarette smoking cessation by e-cigarette use status. Dots represent odds ratio and horizontal lines represent 95% confidence intervals. *P < 0.05; **P < 0.01; ***P < 0.001; aOR = adjusted odds ratio; CI = confidence interval; EC = e-cigarette; Ref = reference.

Fig 3 shows the results of multivariate analyses between e-cigarette use and each stage in smoking cessation controlling for significant covariates. Both current and former e-cigarette users were significantly more likely to be in the PC (current: aOR = 1.44, 95% CI = 1.02–2.03; former: aOR = 1.29, 95% CI = 1.02–1.65) and C stages (current: aOR = 1.60, 95% CI = 1.03–2.48; former: aOR = 1.90, 95% CI = 1.40–2.57), but not to the P and A stages. Current users have highest odds of being A stage, whereas were significantly less likely to be in the M stage (aOR = 0.31, 95% CI = 0.13–0.74) compared to never users. Former users showed that the likelihood of each stage of quitting smoking decreased after the C stage. They have lower odds of being in the M stage (aOR = 0.98, 95% CI = 0.10–0.98) than never users, although it is non-significant. The raw values with CIs are listed in the S2 Table.

## Discussion

This study investigated the relationship between e-cigarette use (i.e., current, former, and never use) and cigarette quitting behavior using all stages of the SOC model. Through the investigation, we could reveal that the SOC model provides detailed and comprehensive information on the role of e-cigarette use in smoking cessation. E-cigarette use was positively associated with past quit attempts, while not with being quitter, intention to quit, and longer duration of quitting. Based on the cessation stages, e-cigarette use may be closely linked with the early-stage but not with late-stage in the entire process of smoking cessation: both current and former e-cigarette users were more likely to be in the PC and C stages, while not to be in the P and A stages, and particularly current users were less likely to be in the M stage than never users. The following paragraphs will discuss the potential positive and negative role of e-cigarette use in smoking cessation process based on our findings. The terms used in this discussion for a specific pattern of e-cigarette use (e.g., dual use, complete switching, and non-current use) can referred to the Fig 1.

Our study found that current e-cigarette users were significantly more likely to be in the PC and C stages, but not in the P stage, compared to never users. This finding suggests that e-cigarette use in smokers (i.e., dual use) was highly related to past quitting attempts but not to strong intention, even among recent quit attempters. This is inconsistent with previous researches including a prior study on Korean adults, which reported that e-cigarette use is related to the stronger readiness to quit smoking (i.e., PC < C < P stages) [27]. We further raise the possibility that such a positive relationship between e-cigarette use and the P stage in previous works may be the effect of past quitting attempts evaluated only in the P stage. Considering that more than two-thirds (69.9%) of dual users are in the NA or PC stages, dual use is no longer indicates a greater readiness to quit smoking in adult smokers. Our results support the claims that dual use may promote continued smoking [40, 41].

A recent study stated that the cessation term is one of the important indicators for estimating the effectiveness of e-cigarette use [42]. Our results verify this, suggesting that current e-cigarette users were significantly less likely to be quitters (i.e., complete switching) when quitting period was ignored, but had the highest odds to be in the A stage and significantly less likely to be in the M stage in the SOC model when a 6-month quitting period was considered. These results imply that e-cigarette use may contribute to short-term (≤ 6 months) quitting as reported in previous studies [12, 14]. On the other hand, we also suggest that the positive relevance of e-cigarette use with quitting smoking may be limited to a short period (≤ 6 months). Indeed, one cohort study showed that the effect of e-cigarette use on smoking cessation was significant only for 6-month, not for 12-months or 18-months of smoking abstinence [29]. Future works will require tracking abstinence for at least longer than six months since completely switching from cigarette to e-cigarette and better if the results were compared across the multiple periods of abstinence.

The negative association between current e-cigarette use and the M stage can be interpreted as both positive or negative impacts of e-cigarette use on long-term (>6 months) quitting: the former is that current e-cigarette users in the A stage also stopped using e-cigarettes (i.e., non-current use of either cigarettes or e-cigarettes) in the M stage; the latter is that they relapsed to current smoking (i.e., dual use or exclusive smoking with e-cigarette use history) before reaching the M stage. Meanwhile, our other results for former e-cigarette users provide important clues for comparing the magnitude of these two possible scenarios; former e-cigarette users were slightly less likely to be in the M stage (i.e., non-current use of either cigarettes or e-cigarettes), and significantly concentrated in the PC and C stages (i.e., exclusive smoking with e-cigarette use history) than never users. These infer that some e-cigarette users may achieve long-term success in quitting cigarettes, while most return to cigarette smoking with no more have intention to quit within the next month.

Collectively, e-cigarette use may be positively related to making quit attempts and possibly to short-term quitting, but more strongly related to current smoking with no immediate intention for future attempts. Due to the difficulty of quitting, smokers generally repeated the short-term abstinence and failed quit attempts [43]. Unfortunately, these multiple quit attempts do not always result in successful quitting and sometimes negatively affect to smoker’s further motivation to quit smoking [44]. Although we could not distinguish whether e-cigarettes were used in recent quit attempts, the collective set of our results suggest that e-cigarette use is possibly linked with failed quit attempts with a weakened intention to quit. Given our other finding that former e-cigarette users were tended to be heavier smokers than current and never users, these results support the concern that e-cigarettes use may have unintended consequences, including perpetuating nicotine addiction and reducing motivation to quit smoking [17, 40, 45]. In this regard, the high prevalence of e-cigarette use among adult smokers will not contribute to a decrease in smoking rate at the population level.

From the perspective of SOC, traditional outcome measures of smoking cessation are sensitive only to transition from the P to the A stages (i.e., making a quit attempts) or the A to the M stages (i.e., smoking abstinence) [18]. Compare to the binary cessation outcomes, the SOC model has a unique power to detect the initial changes in current smokers, and long-term failure of cessation [18]. Given that majority of e-cigarette users no more have a strong intention to quit smoking than never users, more attention is needed on e-cigarette users’ intentional changes for quitting smoking. Moreover, long-term failures are diluted by short-term success in quitting, so binary cessation outcomes without sufficiently long observational periods may overestimate the positive effects of e-cigarette use on quitting smoking. Therefore, the full SOC model can be a valuable alternative to cessation outcomes for measuring the efficacy of e-cigarette use in quitting smoking in the real-world setting [46, 47]. In particular, the current study proposed an extended SOC model reflecting prior quit attempts, complementing the heterogeneity of past quit attempts in the PC and C stages in the traditional SOC model. Applying this expanded SOC model makes it possible to designate smokers who have attempted to quit within the past year but no longer plan to quit smoking immediately. As for e-cigarette users, such stagnant status contains important clues about their ‘sustained dual use’ and ‘smoking relapse’. Therefore, our results using this extended SOC model will provide insights for future empirical studies tracking the cessation behavior of e-cigarette users.

The effectiveness of e-cigarettes in quitting smoking is one of the determinants of their overall public health impact [4]. To maximize the positive impact of e-cigarette use on smoking cessation at the population level, completely switching from cigarette to e-cigarettes rather than dual use and, ultimately, cessation of both products should be encouraged. Dual users who even have no immediate intention to quit smoking can rather increase the negative impact, so their proportion in the total e-cigarette users and the underlying motivation for use should be investigated in the future.

There are several limitations to this study. First, due to the cross-sectional nature of the data, we could not determine the temporal context between e-cigarette use and smoking cessation outcomes. Even though, our large-scale nationwide cross-sectional analysis provides population-level insight into the quitting status of e-cigarette users [11]. Second, we could not adjust for other e-cigarette-related factors, such as the frequency or duration of e-cigarette use, motivation for use, or the nicotine levels in e-cigarette solutions, because the information was not available. To determine the health effects of e-cigarette use, further research is needed to estimate changes in total consumption of nicotine among dual users of e-cigarettes and conventional cigarettes, considering their initial reason for use. Third, the small cell counts (i.e., 14) for the M stage in current e-cigarette users may have potential overfitting with high variance of the estimates. Given this statistical issue, we do not completely rule out the potential for a positive relationship between e-cigarette use and long-term quitting in the discussion. Lastly, we excluded the long-term (>2 years) quitters in the analysis, so the results could be limited to explain the relationship between e-cigarettes and success in quitting more than two years. Nevertheless, since quitters with a longer abstinence period may be less likely to use this relatively new product, restricting participants to those who had quit within recent years could effective in preventing overestimation of the negative relationship between e-cigarette use and long-term quitting.

## Conclusion

The SOC model provided detailed and comprehensive information on the role of e-cigarette use in the entire smoking cessation process. E-cigarette use may be positively related to quitting attempts and possibly to short-term quitting, but is more strongly related to current cigarette smoking with no immediate intention to quit smoking for future attempts. These results imply that e-cigarette use may promote smoking cessation in some smokers, but the negative role of inducing dual use or continued smoking appears to be dominant in the real world at the population level.

## Supporting information

S1 Fig Percentage of sustained cessation over the quitting duration by e-cigarette use status. This is a plot displaying unadjusted percentages of sustained quit (event) over the quitting period (time) according to e-cigarette use status. The estimates were computed using SAS PROC LIFETEST with the Kaplan-Meier method. The small circle indicates the percentage of sustained quitting at 2 years (i.e., cut-off values of ‘recent quitters’ in this study) of current, former, and never e-cigarette users. The values can be calculated as the number of quitters who have sustained cessation for more than 2 years divided by the total number of quitters at the initiation time.
(TIF)

S2 Fig The expanded classification of the stages of change in smoking cessation that reflects quit attempts. The stages of change (SOC) in smoking cessation cover the four quitting-related factors: (i) current behavior (current smokers; recent quitters); (ii) past-year quit attempt (yes; no), (iii) intention to quit (within the next 1 month; within the next 6 months; not within the next 6 months) and (iv) duration of quitting (6 months or less; more than 6 months). Compared to the traditional SOC model, the model proposed in this study is expanded to reflect past-year quit attempts. We isolated individuals who had not made a quit attempt in the past year from the ‘Precontemplation’ (PC) and ‘Contemplation’ (C) stages and set them as the ‘No attempt’ (NA) stage for the expanded SOC model.
(TIF)

S1 Table Prevalence of cessation stages by e-cigarette use status. Values are unweighted frequencies (N), weighted percentages (%), and P-values according to chi-square test. EC = e-cigarette.
(DOCX)

S2 Table Associations between e-cigarette use and current quitting behavior and the stages of change in smoking cessation (N = 3,929). Multivariate models were adjusted for all covariates and survey year. *P < 0.05; **P < 0.01; ***P < 0.001; Bold type P < 0.05. aOR = adjusted odds ratio; CI = confidence interval; EC = e-cigarette; Ref = reference.
(DOCX)

## Abbreviations

A: action
C: contemplation
CI: confidence intervals
e-cigarette: Electronic cigarette
M: maintenance
NA: No attempt
OR: odds ratios
P: preparation
PC: pre-contemplation
the SOC model: the stages of change model
