# Supplementary figures and images for: Understanding the role of e-cigarette use in smoking cessation based on the stages of change model
Source: PLoS One. 2022 Sep 9;17(9):e0274311. doi: 10.1371/journal.pone.0274311 (PMC9462758; doi:10.1371/journal.pone.0274311)

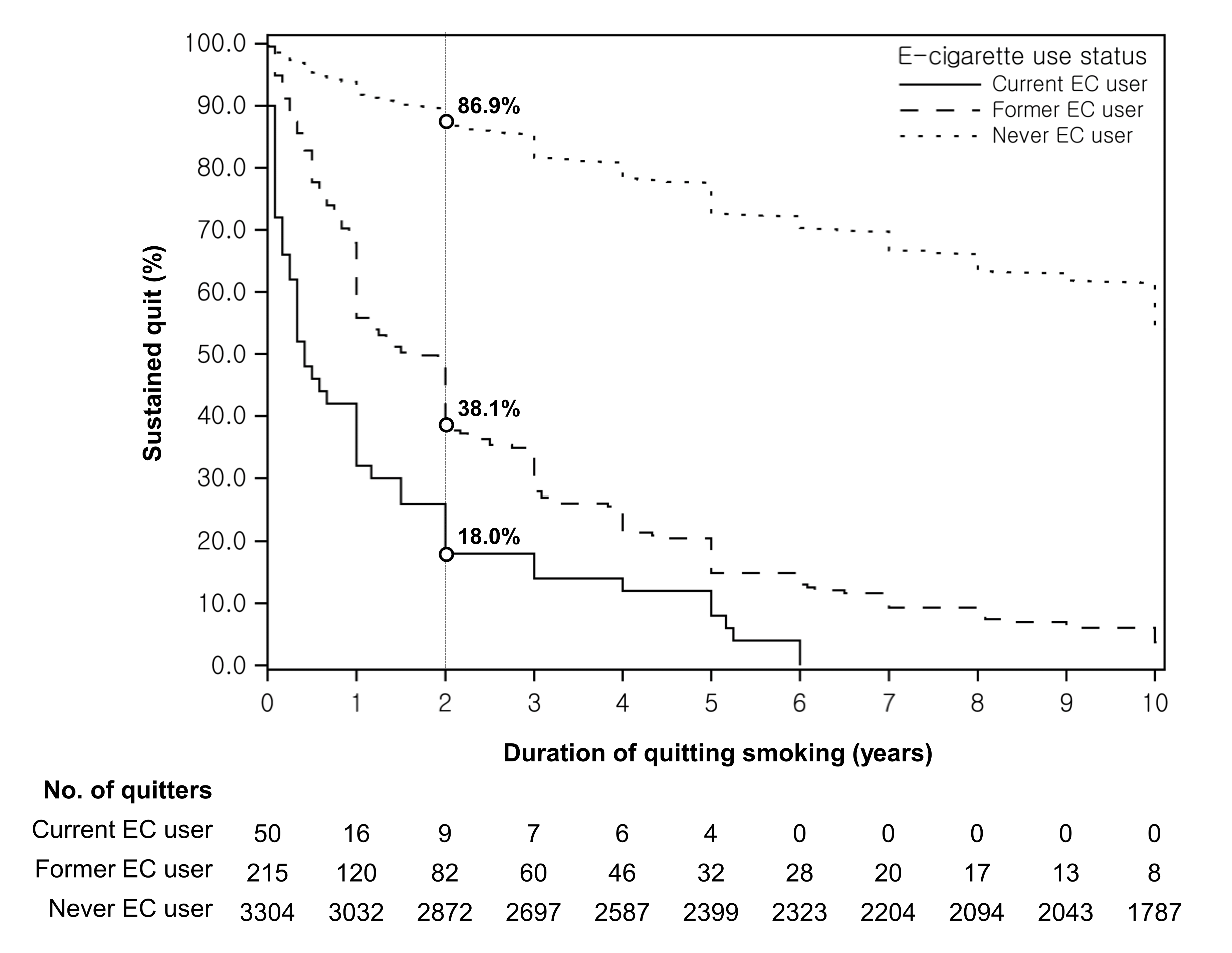

Supplement: S1 Fig — This is a plot displaying unadjusted percentages of sustained quit (event) over the quitting period (time) according to e-cigarette use status. The estimates were computed using SAS PROC LIFETEST with the Kaplan-Meier method. The small circle indicates the percentage of sustained quitting at 2 years (i.e., cut-off values of ‘recent quitters’ in this study) of current, former, and never e-cigarette users. The values can be calculated as the number of quitters who have sustained cessation for more than 2 years divided by the total number of quitters at the initiation time. (TIF) [file pone.0274311.s001.tif]

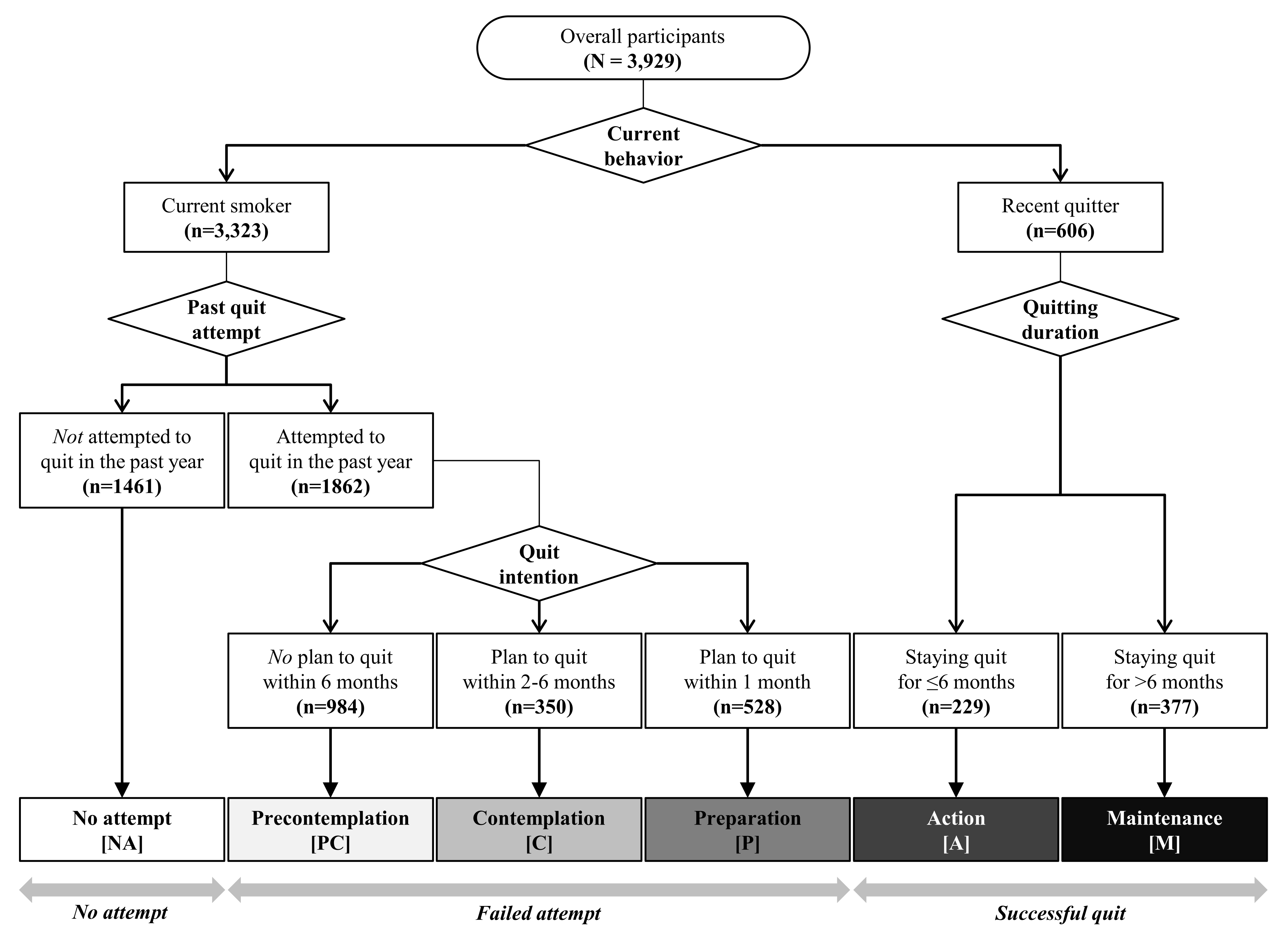

Supplement: S2 Fig — The stages of change (SOC) in smoking cessation cover the four quitting-related factors: (i) current behavior (current smokers; recent quitters); (ii) past-year quit attempt (yes; no), (iii) intention to quit (within the next 1 month; within the next 6 months; not within the next 6 months) and (iv) duration of quitting (6 months or less; more than 6 months). Compared to the traditional SOC model, the model proposed in this study is expanded to reflect past-year quit attempts. We isolated individuals who had not made a quit attempt in the past year from the ‘Precontemplation’ (PC) and ‘Contemplation’ (C) stages and set them as the ‘No attempt’ (NA) stage for the expanded SOC model. (TIF) [file pone.0274311.s002.tif]
