# Supplementary material for: Understanding the role of e-cigarette use in smoking cessation based on the stages of change model
Source: PLoS One. 2022 Sep 9;17(9):e0274311. doi: 10.1371/journal.pone.0274311 (PMC9462758; doi:10.1371/journal.pone.0274311)
Supplement: S1 Table — Values are unweighted frequencies (N), weighted percentages (%), and P-values according to chi-square test. EC = e-cigarette. (DOCX) [file pone.0274311.s003.docx]

**S1 Table. Prevalence of cessation stages by e-cigarette use status.**

|  | Current EC user  (N = 353) | | Former EC user  (N = 881) | | Never EC user  (N = 2,695) | |  |
| --- | --- | --- | --- | --- | --- | --- | --- |
|  | N | % | N | % | N | % | *P* |
| Stages of change |  |  |  |  |  |  |  |
| No attempt | 128 | 34.8 | 302 | 34.8 | 1031 | 38.9 | <0.001 |
| Precontemplation | 88 | 27.9 | 233 | 25.2 | 663 | 24.2 |  |
| Contemplation | 42 | 11.4 | 111 | 13.9 | 197 | 7.7 |  |
| Preparation | 54 | 14.4 | 102 | 11.5 | 372 | 13.6 |  |
| Action | 27 | 7.8 | 48 | 5.3 | 154 | 5.4 |  |
| Maintenance | 14 | 3.7 | 85 | 9.3 | 278 | 10.1 |  |

Values are unweighted frequencies (N), weighted percentages (%), and P-values according to chi-square test.

EC = e-cigarette.
