# Supplementary material for: Understanding the role of e-cigarette use in smoking cessation based on the stages of change model
Source: PLoS One. 2022 Sep 9;17(9):e0274311. doi: 10.1371/journal.pone.0274311 (PMC9462758; doi:10.1371/journal.pone.0274311)
Supplement: S2 Table — Multivariate models were adjusted for all covariates and survey year. *P < 0.05; **P < 0.01; ***P < 0.001; Bold type P < 0.05. aOR = adjusted odds ratio; CI = confidence interval; EC = e-cigarette; Ref = reference. (DOCX) [file pone.0274311.s004.docx]

**S2 Table. Associations between e-cigarette use and current quitting behavior and the stages of change in smoking cessation (N = 3,929).**

|  | Stages of change in cigarette smoking cessation (vs. No attempt) | | | | | | | | | |
| --- | --- | --- | --- | --- | --- | --- | --- | --- | --- | --- |
|  | Precontemplation | | Contemplation | | Preparation | | Action | | Maintenance | |
|  | aOR | (95% CI) | aOR | (95% CI) | aOR | (95% CI) | aOR | (95% CI) | aOR | (95% CI) |
| E-cigarette use status |  |  |  |  |  |  |  |  |  |  |
| Current EC user | **1.44*** | **(1.02–2.03)** | **1.60*** | **(1.03–2.48)** | 1.45 | (0.94–2.24) | 1.65 | (0.92–2.93) | **0.37**** | **(0.20–0.70)** |
| Former EC user | **1.29*** | **(1.02–1.65)** | **1.90***** | **(1.40–2.57)** | 1.14 | (0.85–1.53) | 1.10 | (0.72–1.69) | 0.98 | (0.71–1.35) |
| Never EC user | 1.00 | Ref. | 1.00 | Ref. | 1.00 | Ref. | 1.00 | Ref. | 1.00 | Ref. |
|  | *P* = 0.0001 | | | | | | | | | |

Multivariate models were adjusted for all covariates and survey year. *P < 0.05; **P < 0.01; ***P < 0.001; Bold type P < 0.05. aOR = adjusted odds ratio; CI = confidence interval; EC = e-cigarette; Ref = reference.
